# Errors in fracture diagnoses in the emergency department – characteristics of patients and diurnal variation

**DOI:** 10.1186/1471-227X-6-4

**Published:** 2006-02-16

**Authors:** Peter Hallas, Trond Ellingsen

**Affiliations:** 1 Department of Surgery, Hålogalandssykehuset Harstad, 9406 Harstad, Norway

## Abstract

**Background:**

Evaluation of the circumstances related to errors in diagnosis of fractures at an Emergency Department may suggest ways to reduce the incidence of such errors.

**Methods:**

Retrospective analysis of all cases during a two year period (2002–2004) where a fracture had been overlooked or an injury had been erroneously diagnosed as a fracture (n = 61). 100 random selected patients with correctly diagnosed fractures served as control group.

**Results:**

In the two year period 5879 patients visited the ED with injuries. 1% of all visits to the ED resulted in an error in fracture diagnosis and 3.1% of all fractures were not diagnosed at the initial visit to the ED. 86% of such errors had consequences for treatment. No patient characteristics could be identified as risk factors for a misdiagnosis of a fracture. There was a peak in errors in fracture diagnoses between 8 pm and 2 am (47% against 20% in controls, p < 0.005).

**Conclusion:**

A considerable number of fractures were not correctly diagnosed at the initial ED visit. There was a diurnal variation in the rate of misdiagnosis of fractures with a significant peak from 8 pm to 2 am. Where there was an error in fracture diagnosis, the patients did not appear to have a characteristic profile as regarding e.g. age, sex or capability to communicate with the ED staff. Increased consultancy service in radiology may reduce the frequency of errors in diagnosis, particularly in the evenings between 8 pm and 2 am.

## Background

Misdiagnosis of a fracture is a very common occurrence in Emergency Departments (ED) and can have serious consequences because of delays in treatment and resulting long-term disability [1]. Analysis of the circumstances where errors in medical practice takes place may suggest ways to prevent them.

Studies analysing errors in fracture diagnosis have focused on the nature of the fractures and the interpretation of the x-rays [1-3]. These studies have mentioned the importance of training and supervision of junior doctors and thorough clinical examination as ways of reducing errors in the ED [1,2].

So far no study has examined whether patients who experience a diagnostic error have any common characteristics or whether there is any diurnal pattern in the rate of errors in fracture diagnosis as seen in other studies of diagnostic tests [3].

In the light of this we present a retrospective analysis of all errors in fracture diagnosis in patients who visited the ED or were admitted to the Department of Orthopaedics, Harstad Hospital, Norway, during a two year period.

## Methods

The ED at Harstad Hospital serves a population of 43,000 in the Arctic region of Norway. All patients with injuries who present to the ED are seen by an intern from the Department of Surgery. The intern can always ask a resident for advice in cases of doubt. A consultant in radiology reviews the x-rays the next morning (except in weekends and on public holidays) and the patient is contacted if the diagnosis is changed.

Cases of errors in fracture diagnosis were identified by reviewing all diagnoses of patients who visited the hospital from 1.5.2002 to 1.5.2004, using the hospitals electronic patient file system to identify cases [4].

Medical records were reviewed if a patient was diagnosed with a fracture and at least one other injury during the two year period. Cases were included if the review of the medical record revealed that a fracture was missed on the initial visit to the ED or if an injury was erroneously diagnosed as a fracture on the initial visit. The diagnosis made by a consultant in radiology was considered gold standard.

Not included were patients with rib fractures or scaphoid fractures unless the fractures were visible on x-rays on the initial visit. Patients were not included if the radiologist expressed doubt about the correct diagnosis.

A control group consisting of 100 patients was randomly selected from all patients who were correctly diagnosed with a fracture on their first visit to the ED during the two year period. For information on sex, date of visit and type of fracture all patients with correctly diagnosed fractures in the two year period were used as controls. Data on time of treatment was available from the x-rays (it is noted on the film what time the x-ray is taken) or in some cases from the medical record. Calculations of the percentage of missed fractures were done for each hour of the day to be able to identify any diurnal variations.

Since x-rays can not be identified separately in the DIPS system, the total number of true negatives could not be established, thus limiting the opportunities for calculating specificity.

Chi-test and the Normal Test were used for statistical analysis. A significance level of P < 0.05 was used for all tests.

## Results

In the two year period 5879 patients visited the ED with injuries and 1323 were treated for a fracture. 40 patients had fractures that were not diagnosed on the initial visit (false negative diagnoses) and 21 patients were erroneously diagnosed with a fracture on the initial visit (false positive diagnoses). Thus 1% of all visits to the ED results in an error in fracture diagnosis and 3.1% of all fractures were not diagnosed at the initial visit to the ED.

The missed fractures were in the ankle or foot (28%, n = 11), lower arm (22%, n = 9), hand and fingers (22%, n = 9), hip (10%, n = 4) and misc. (18%, n = 7). This anatomical distribution of fractures did not differ significantly from that in the control group (p > 0.05). Data on patient characteristics and x-ray interpretation are presented in table 1. One patient had dementia (NS), one was alcohol intoxicated (NS) and language barrier problems were noted in the medical records of six patients (NS).

**Table 1:** Patient characteristics and results of x-ray interpretation

Fracture diagnoses (2002–2004)	False Positive	False negative	Sum Diagnostic Errors	Control group	P
N	21	40	61	100	
Male: Female	8:13	19:21	27:34	50:50	NS
Age Yrs (s. d.)	31 (20.6)	45.1 (27.7)	40.2 (26.2)	44.7 (27.3)	NS
First ED visit between 8 pm – 2 am*	13	13	26	20	<0.005
X-ray on first ED visit	21	33	54	99	<0.005
Doubt of x-ray interpretation noted by intern	13	13	26	9	<0.001
Seen only by an intern	14	23	37	86	NS
Intern + a resident	4	14	18	10	<0.05
Intern + a consultant (surgery or radiology)	3	3	6	1	NS

*Digits not available for all patients

Data on time of visit was not available for six patients (10%) with false negative fracture diagnosis because they did not have an x-ray taken on the first visit to the ED and no time was noted in the medical record. In the case of one patient in the control group it was not necessary to confirm the fracture diagnosis with x-ray and no other data on time of visit was available for this patient. Thus data on time of day of the visit to the ED was available for 55 patients and 99 controls.

**Figure 1:**
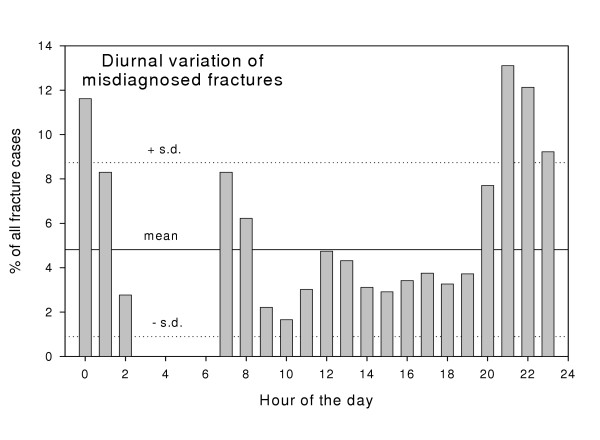
**Diurnal distribution of errors**. Diurnal distribution of errors in fracture diagnosis. 47% of patients who were subjected to mistakes in diagnoses visited the emergency department between 8 p.m. to 2 a.m. compared to 20% of controls (p < 0.005).

The diurnal distribution is shown in fig. 1. 47% of all patients subject to errors in diagnoses visited the ED between 8 pm to 2 am compared to 20% of controls (p < 0.005) (table 1). The positive predictive value of a fracture diagnosis was 99% in the hours between 2 am to 8 pm and 95% in the hours between 8 pm to 2 am. The sensitivity of the x-ray interpretation was 97% between 2 am to 8 pm and 95% in the hours between 8 pm to 2 am. (Specificity could not be calculated because data on time of visit to the ED was not available for patients who had not been diagnosed with a fracture at some point in their treatment). Only 3% of patients had x-rays taken between 2 am to 8 am and during these hours no fractures were missed (NS).

There was no significant difference between week days and weekends in the number of patients with errors in diagnosis. During the Arctic winter and skiing season (the 6 months from November till May) there were on average 242 visits regarding injuries to the ED per month as compared to an average of 248 visits per month for the remainder of the year. In the winter months 24% of all patients with injuries were diagnosed with a fracture compared to 20% during the remainder of the year (p < 0.05), but the percentage of errors was unchanged: 49% of the errors in fracture diagnosis were made during the six months of winter (NS). There were no peaks in errors after the half yearly arrivals of new interns and no single rotation of interns was found to have an increased incidence of errors (NS).

Correct diagnosis was delayed 3.9 days on average (s.d. 0.5; median 1 day). Once the wrong diagnosis was discovered, then in 86% of the cases the treatment was changed. In the majority of the remaining 14% of cases a sufficient treatment had already been instituted even though the fracture had not been diagnosed.

## Discussion

The 1% incidence of missed fractures is comparable to the incidence in other institutions [1-3]. The fact that the majority of errors had consequences for treatment emphasises the importance of reducing the incidence of diagnostic errors.

This study might underestimate the number of errors made because data was retrospectively collected and also because some patients could have been lost to follow-up (although the nearest other hospital is 120 km away through mountainous Arctic country). In addition some doctors might not state information on e.g. language difficulties or advice from senior colleagues in the medical records. Lack of time of visit information for 10% of cases could induce a selection bias in the analysis of diurnal variation so the result should be interpreted with caution.

Contrary to common belief about errors in fracture diagnosis [2], the patients involved were not more demented, older, more difficult to communicate with or more drunk than controls. This means that cases of errors in fracture diagnosis can not as a rule be attributed to factors such as the behaviour of the patient. This again shows the importance of a thorough clinical examination of all patients as a way of reducing errors.

There was no anatomical region where a fracture was especially prone to be misdiagnosed in this study of an ED with a mixed caseload. Others have shown that certain types of fractures are more likely to be misdiagnosed in paediatric ED patients [5]. Thus a focus of such sub-groups of ED patients might help identify fractures that are prone to diagnostic errors.

This study has shown a diurnal variation in the number of patients who are subject to errors in diagnosis, with a significant percentage of errors being made in the evening and overnight (table 1). The fatigue experienced by junior doctors after a night's work is well-known to increase the number of errors in the interpretation of other diagnostic tests, e.g. ECG [6], in medication [7] and in performance of routine procedures [8]. In addition visual vigilance is one of the more sensitive areas to show deterioration as effect of tiredness [9]. The number of errors in this study, however, peak from 8 pm to 2 am rather than increase proportionally during the night. Thus impaired performance due to fatigue of the doctors in the ED is probably not the main explanation for the diurnal variation in diagnostic errors.

When estimating the degree of fatigue it should be noted that the work patterns of interns in Norway are strictly regulated by a collective bargaining agreement limiting the weekly work to a maximum of 40 hours (though exceptions can be made). For most of the study period the interns worked 24-hour shifts. The interns at our institution have a six month rotation in Department of Surgery (which includes taking care of ED patients). The study period involved five groups of an average of 8 interns. Thus the number of interns sharing the shifts and the maximum hours allowed per week for each doctor limits the number of days per month that each intern is on a 24-hour shift.

Since no single rotation was found to have an increased incidence of errors it is unlikely that the diurnal pattern is a result of a small number of poorly performing doctors.

Like in other studies [1,2,5] most of the errors were a consequence of misreading of x-rays. There were frequent indications in the medical records that the doctor was in doubt about the correct interpretation of the x-ray, and errors happened even in the 38% of cases where a senior colleague saw the x-rays and suggested a diagnosis. These findings suggest that, where errors were made in fracture diagnosis, patients often had fractures that were difficult to see on x-ray, e.g. minor injuries. Thus one possible explanation of the diurnal variation of errors might be that patients with minor injuries and subtle factures wait till the evening – when job and housework have been taken care of – before presenting to the ED. A future study examining the interval between time of injury and visit to the ED for patients who are misdiagnosed might help clarify this point. At Harstad Hospital there is no consultancy service in radiology available between 3 pm and 8 am, so there may be insufficiently help at hand for the interpretation of x-rays findings when these patients present to the ED.

Thus it is likely that a combination of factors caused the observed diurnal variation in missed fracture diagnosis: patients with fractures that are difficult to see on x-ray presenting to the ED at a time of day where the doctors on call are tired and there is little help at hand from the radiology department. An additional factor explaining the peak in errors might be that errors rarely occur between 2 am to 8 am simply because there are very few patients (3% of controls) coming to the ED during these hours. Only patients with serious (and thus obvious) injuries may elect to travel through Arctic county during the night to get to the hospital.

How can these observations help reduce the number of errors in fracture diagnosis?

Error rates can be reduced to below 0.3% by changes such as increased co-operation between emergency physicians and radiologists and the introduction of a training file of radiographs [3]. Could error rates be even further reduced by focusing on possible causes of the diurnal pattern? The work schedule for junior doctors has now been changed in our department by dividing the day into a day shift and a night shift. No doubt increased availability of a consultancy service would help with the interpretation of difficult x-rays [3] but in a small hospital in a remote area like ours it would not be possible to have a consultant in radiology on in-house-call. Teleradiology is safe and effective [10,11] in the diagnosis of fractures and might in the future prove a method of providing consultancy service in radiology to remote settings.

## Conclusion

A considerable part of all fractures were missed at the initial ED visit. There is a peak in mistakes in fracture diagnosis in the ED between 8 pm and 2 am. This diurnal variation might occur because patients with subtle fracture or minor injuries present at the end of their working day and after any home duties have been dealt with. Increased access to consultancy service in radiology might reduce the number of errors in diagnosis.

## Competing interests

The author(s) declare that they have no competing interests.

## Authors' contributions

PH conceived of the study, gathered and analysed data and drafted the manuscript. TE supervised the project and participated in the interpretation of the data.

Both authors read and approved the final manuscript.

## Pre-publication history

The pre-publication history for this paper can be accessed here:


